# Intersections of Intimate Partner Violence and Natural Disasters: A Systematic Review of the Quantitative Evidence

**DOI:** 10.1177/15248380241249145

**Published:** 2024-05-21

**Authors:** Jennifer Boddy, Celeste Harris, Patrick O’Leary, Madeleine Hohenhaus, Christine Bond, Christopher Panagiotaros, Leah Holdsworth

**Affiliations:** 1 Griffith University, QLD, Australia; 2 Climate Action Beacon, Griffith University, QLD, Australia; 3 Disruption Violence Beacon, Griffith University, QLD, Australia

**Keywords:** natural disasters, intimate partner violence, systematic review

## Abstract

Natural disasters and extreme weather events are increasing in both intensity and frequency. Emerging evidence suggests that there is a relationship between intimate partner violence (IPV) and natural disasters. However, there is a scarcity of methodologically sound research in this area with no systematic review to date. To address the gap, this paper systematically assesses the quantitative evidence on the association between IPV with natural disasters between 1990 and March 2023. There were 27 articles that meet the inclusion criteria for the data extraction process. A quantitative critical appraisal tool was used to assess the quality of each study and a narrative synthesis approach to explore the findings. The review found an association between IPV and disasters, across disaster types and countries. However, more research is needed to explore the nuances and gaps within the existing knowledge base. It was unclear whether this relationship was causal or if natural disasters heightened existing risk factors. Further, it is inconclusive as to whether disasters create new cases of IPV or exacerbate existing violence. The majority of studies focused on hurricanes and earthquakes with a dearth of research on “slow onset disasters.” These gaps represent the need for further research. Further research can provide a more thorough understanding of IPV and natural disasters, increasing stakeholders’ ability to strengthen community capacity and reduce IPV when natural disasters occur.

## Introduction

Intimate partner violence (IPV) has often been viewed as being exacerbated by the occurrence of natural disasters. Expected increases in frequency and severity of natural disasters, due to the growing impacts of climate change, may mean that there are more instances of IPV, with concurrent disruptions to support services for individuals and families. Natural disasters continue to increase in intensity and frequency and, due to the lack of actions and policies by nations globally, will continue to do so (IPCC, 2022; Perkins, 2017). Globally, there is a growing body of research (both qualitative and quantitative) exploring the relationship between extreme weather events and IPV. This expanding, international literature has highlighted the need to accurately quantify the magnitude of this issue (Gearhart et al., 2018) and rigorously study patterns of violence associated with natural disasters (Rezaeian, 2013). However, this has yet to happen in a systematic way. The lack of a sound evidence base to substantiate the link between IPV and natural disasters creates a barrier to effectively preventing and responding to these crises.

This systematic review maps the quantitative evidence base examining natural disasters and IPV in order to guide future research by: (a) understanding the association between the exposure to natural disasters and the experience of IPV perpetrated against adult women; (b) determining the quality of this evidence; and (b) identifying gaps in the evidence base to guide further studies. It builds on the systematic review by Rezaeian (2013), who surveyed the literature on violence and natural disasters, published between 1978 and 2011. Using a health search engine (PubMed), the author found 21 original articles, just under half (*n* = 10) on the relationship between natural disasters and interpersonal violence (including IPV). Overall, Rezaeian (2013) found that, with one exception, these studies suggested that exposure to natural disasters was associated with increased gendered violence. However, the review concluded that there was a scarcity of methodologically sound research in this area at that time, recommending further studies measuring the magnitude/patterns of violence across natural disasters and the “effects of any possible moderating or confounding variables, e.g., age, sex, income, family and social” (p. 3). Thus, 10 years later, it is timely to assess what we know about the association between natural disasters and IPV, especially given the limited results of Rezaeian’s search.

### Natural Disasters and IPV

Natural disasters are catastrophic events triggered by naturally occurring hazardous phenomena, with geophysical, meteorological, hydrological, climatological, biological, or extraterrestrial origins (Below et al., 2009). These hazards become disasters due to their interaction with social systems and vulnerable communities (Sewell et al., 2016), resulting in severe adverse consequences including loss of life and injury, loss of property, environmental damage, and long-term social and economic costs to communities. Globally, natural disasters are increasing in severity (Gearhart, et al., 2018), resulting in substantial increases in the economic costs to communities. The estimated economic loss from natural disasters between 1998 and 2017 was approximately US$2,908 billion, a 68% increase in the estimated costs from the previous two decades (1978–1997) (Centre for Research on the Epidemiology of Disasters & United Nations Office for Disaster Risk Reduction, 2017).

While natural disasters disrupt both physical and social environments (Rodriguez et al., 2007), early disaster research found that disasters of similar nature and magnitude had dramatically different consequences for people in different locations across the globe (O’Keefe et al., 1976). Researchers have explained these differential consequences between individuals, groups, communities, and countries as a function of their level of vulnerability (Zakour & Gillespie, 2013, p. 18). Known as disaster vulnerability theory, this perspective links increased vulnerability, which is a direct result of unsafe physical and social conditions (Wisner et al., 2004) to a higher probability of a disturbance from a disaster, greater severity of the experience, as well as a poorer post-disaster response (Zakour & Gillespie, 2013).

Individuals’ vulnerability is also related to their social characteristics. Certain characteristics reduce or increase their vulnerability to adverse outcomes after disasters. In particular, gender has been shown to increase vulnerability across all ages and cultures (Bolin, 2007). Females experience more severe consequences from disasters than males, across a range of health and social outcomes, with a growing number of studies documenting increases in women’s harmful experiences of interpersonal violence, sexual violence, and IPV following disaster events (Gearhart, et al., 2018; Norris et al., 2002).

Increasing incidents of IPV—the physical, sexual, psychological, and/or emotional abuse inflicted by an intimate partner—after disasters is particularly concerning. IPV is strongly gendered, with most perpetrated by males against females (Mitchell, 2011; Phillips & Vandenbroek, 2014), and is one of the most common forms of violence against women across the globe (World Health Organization, 2012). IPV has been identified as a global public health issue and serious abuse of human rights (Coll et al., 2020; Garcia-Moreno et al., 2006), given its severe impacts on the physical, psychological, sexual, and reproductive health of women and families (Coll et al., 2020).

Across the globe, almost 30% of women have experienced physical and/or sexual IPV and approximately 38% of female homicide victims are murdered by an intimate partner (World Health Organization 2021). Large economic costs are also associated with IPV, due to an increased burden on healthcare and service systems, lowered productivity, reduced income for women and families, and decreased future human capital from intergenerational impacts (Duvvury et al., 2013). In the U.S. alone, it has been estimated that IPV results in an economic loss between $1.7 billion and $10 billion annually (Modi et al., 2014).

### Objective

This review systematically assesses the evidence on the associations between IPV during and following natural disasters. As the focus is on the association between exposure to natural disasters and IPV, the review only includes quantitative study designs and original research.

## Methods

### Design

To identify and synthesize the available quantitative evidence on the association between natural disasters and IPV, we undertook a systematic literature review. At the time of writing this paper, we were not aware of any other systematic reviews on this topic. The review adheres to the PRISMA (Preferred Reporting Items for Systematic Reviews and Meta-analyses) guidelines (Moher et al., 2009). Due to heterogeneity of the results and diversity in outcome measures in the identified studies, a meta-analysis was not possible. As a result, this review uses the reporting protocol of synthesis without meta-analysis (SWiM) (Campbell et al., 2020).

### Eligibility Criteria

A set of inclusion and exclusion criteria was developed to guide the selection of studies relevant for review. As shown in Table 1, the review was limited to: (a) peer-reviewed studies; (b) published between 1990 to March 2023; (c) English language publications; (d) all geographic locations; (e) focused upon natural disasters and IPV; (f) participants were adults, over the age of 18; and (g) original quantitative research measuring changes in, or association between, IPV and natural disaster/s (change includes magnitude and/or severity).

**Table 1.** Search Terms and Review Criteria.

Search Terms	
Category 1. intimate partner violence, domestic violence (DV), domestic abuse, violence against women, domestic and family violence, gender based violence, spous* assault, spous* abuse, battered women, partner violence, partner abuse. Category 2. natural disaster, post-disaster, post disaster, extreme weather, bushfire, wildfire, flood, storm, hurricane, earthquake, tornado, heatwave, heat wave, tsunami, tidal wave, drought, cyclon*, typhoon, volcano, volcanic eruption, blizzard, avalanche, landslide, typhoon.	
Inclusion Criteria	Exclusion Criteria
- Measured changes in, or association between intimate partner violence (IPV) or DV and natural disaster/s (original quantitative research) - Participants were adults, over the age of 18. - Peer-reviewed - Published from 1990 to March 2023 - English language articles - Occurring in any location worldwide	- Focused on technological or human-caused disasters, such as oil spills and nuclear failures, or biological disasters such as pandemics and epidemics - Focused upon violence outside the scope of IPV (e.g., child abuse, family violence excluding IPV or DV) - Not original peer-reviewed research (reviews, opinion and correspondence articles, book chapters, conference and meeting proceedings)

For the purposes of this review, natural disasters are those triggered by hydrometeorological, geophysical, and climatological events (Below et al, 2009), including both slow and rapid onset disasters. We excluded biological disasters such as epidemics and pandemics and human-caused disasters, such as wars, nuclear failures, or oil spills.

IPV could take the form of physical, sexual, psychological, and/or emotional violence inflicted by an intimate partner (World Health Organization, 2012). Violence inflicted upon or by children or young people, elder abuse, or violence inflicted by/upon any other household member was excluded. Appropriate search terms were then identified, drawing on other systematic reviews (Bell & Forketh, 2016; Cerna-Turoff et al., 2019) (see Table 1).

### Information Sources and Search Strategy

**Figure 1.**
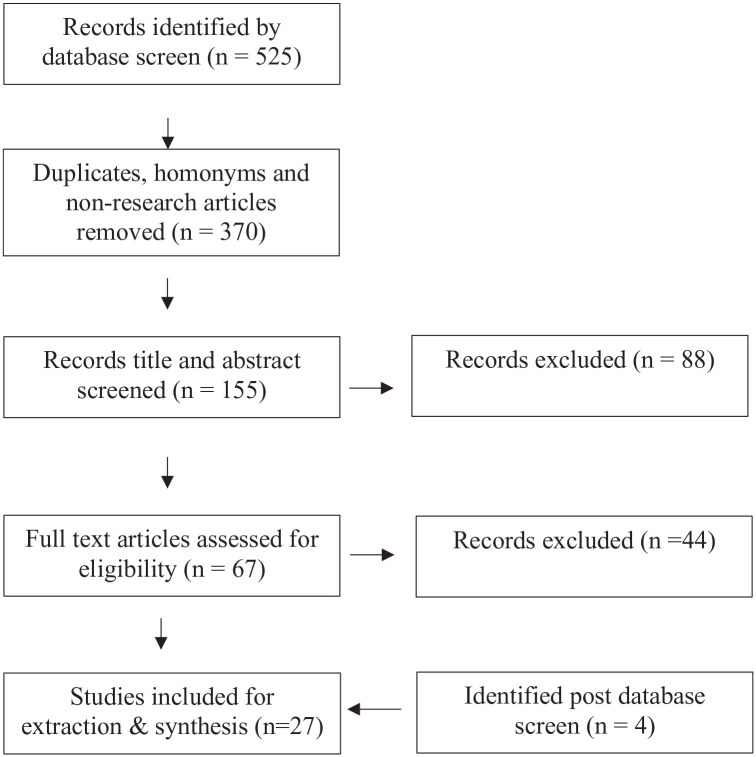
Preferred Reporting Items for Systematic Reviews and Meta-Analyses diagram.

Five academic databases (ProQuest, Web of Science, Scopus, PsycINFO, and PubMed) were searched for articles published between 1990 and March 2023. The search combined search terms from both categories (see Table 1) in the title and the abstract and was repeated for all databases. Boolean logic was used in all searches. Where possible, article type was restricted to scholarly articles. Electronic searches were first conducted in February 2021 and were then replicated in March 2023 to ensure currency. Endnote software was used to manage returned citations (*n* = 439). Duplicate records (*n* = 229), articles including search terms but unrelated meaning (homonyms) (*n* = 36), and nonresearch articles (*n* = 43) were eliminated by reviewing titles/abstracts. Two members of the research reviewed the titles and abstracts of the remaining citations (*n* = 131) in an unblinded, standardized manner using the inclusion and exclusion criteria. The percentage of inter-rater agreement was 71%. Where there was disagreement and uncertainty about inclusion, the full text was screened for eligibility (*n* = 67). A further 44 studies were excluded as they did not meet the eligibility criteria post full text screening, resulting in 23 articles for extraction and synthesis. Four studies, meeting the inclusion criteria, were identified after the database research searches. As they met the inclusion criteria, they were added for extraction. An additional search was undertaken in March 2023 to ensure relevancy, with 86 additional results of which (*n* = 24) abstracts reviewed and (*n* = 62) articles eliminated based on titles/abstracts. As shown in Figure 1, this resulted in 27 articles for inclusion in the data extraction process.

### Quality Appraisal

A total of 27 studies were eligible for inclusion and data extraction. Of these, three articles were case studies with quantitative components and were retained. These three case studies were appraised with the Joanna Briggs Institute (JBI) Model for Evidence-Based Healthcare JBI qualitative research tool, to provide an appropriate and representative score of the study quality. Two studies used mixed methods. These studies were appraised with the Mixed Methods Appraisal Tool (MMAT) (Hong et al., 2018). Twenty-two studies were appraised using the (JBI) checklist for analytical cross-sectional studies.

### Data Extraction

Two research team members developed a data extraction sheet (based on the Cochrane Consumers and Communication Review Group’s data extraction template and in line with SWiM protocols), which was then pilot tested on three randomly selected included studies and modified accordingly. The final extraction table was refined to include relevant information for each article including author and year, study aim, disaster type, number of disasters, disaster date/s, study location, sample size, population, measure of IPV, measure of natural disaster, IPV outcomes, other identified risk factors, confounding or other variables, study design, theoretical framework, and the JBI critical appraisal score.

A third member reviewed and extracted data from included studies, while another member checked the extracted data. Disagreements were resolved by discussion between the two members; all issues were resolved. We contacted five authors of the identified studies for further information. All responded and one provided numerical data that had only been presented graphically in the published paper.

### Data Synthesis

Although a “standardized metric” (Campbell et al., 2020) was considered for the selected quantitative studies, the diversity in results of available data meant that meaningful outcome categories would be difficult to provide. Further, this review included several qualitative studies with quantitative components and two mixed methods studies. In such circumstances, a narrative synthesis is appropriate. As outlined by the Center for Reviews and Dissemination (CRD) (2009), “narrative and quantitative approaches are not mutually exclusive. Components of narrative synthesis can be usefully incorporated into a review that is primarily quantitative in focus” (p. 45). Thus, a narrative synthesis was used, allowing a deeper exploration of the outcomes.

## Results

Table 2 provides a summary of the key characteristics of the identified studies. We synthesized the results in terms of the theoretical framework adopted; disaster type and countries; type of measures of IPV; and risk factors and associations between IPV and natural disasters. The studies come from a diverse range of countries, examine a range of disaster types and forms of IPV, and identify a range of risk factors. Additionally, Table 3 provides a summary of the type of natural disaster, key risk factors, along with protective factors that could contribute to practice and policy suggestions.

**Table 2.** Summary of Research Findings.

Author Name and Year	Disaster Type	Study Location and Dates	Sample Size	Population	IPV Outcomes	Other Identified Risk Factors	Joanna Briggs Institute (JBI) Quality Score
Allen et al. (2021)	Floods	Kenya Floods occurring in 2006, 2007, 2008, 2012, 2013 and 2014)	2008: 4,903 women 2014: 4,512 women	Women who participated in Demographic and Health Surveys (DHS) and completed domestic violence (DV) module	- 2014 sample experienced decrease in reported intimate partner violence (IPV) compared to 2008 - authors suggest that this may be due to changes in legislation in accordance with the United Nations Sustainable Development Goals to protect women - Increased no. of floods led to increased IPV odds for sexual (increased odds of 1.6) and physical violence (increased odds of 1.91), but not emotional abuse.	- Heavy drinking in partner increased odds by 2.38 in all forms of IPV - Partner working in agriculture (1.25 increased odds)	8 out of 8 (100%)—No report on the number of floods that were experienced
Anastario et al. (2008)	Hurricane (Katrina & Rita)	Louisiana and Mississippi, USA, 2005	194 women	Internally Displaced People (IDPs)—women only, living in trailer parks.	- 91 (46.9%) experienced violence in their lifetime - 34 women experienced post-disaster violence (17.5%) - IPV increased by 5.1% in 2006, to 7.6% in 2007	- Women of color - Married (35.3%), never divorced (26.5%), - Likelihood of IPV increased with age from 18 to 36 and decreased from 37 to 85 - Sleeping problems, appetite dysregulation, low self-esteem, suicidal ideation	100%
Anastario et al. (2009)	Hurricanes (Katrina & Rita)	Mississippi, USA, 2005	2006 sample: 106 2007 sample: 314 Total: 420	IDPs—women only, living in trailer parks.	- The prevalence of recent IPV increased by 5.1%, from 2.5% in 2006 to 7.6% in 2007. - Lifetime IPV significantly increased by 21.9%, from 12.5% in 2006 to 34.4% in 2007. - IPV continued to escalate in the 2 years following displacement	- Increased recent and lifetime rate of IPV sig increased risk for major depressive disorder, suicidal ideation and depressive symptoms	100%
Azad et al. (2014)	Floods	Sirajganj District, Bangladesh, 2006–2011	185 women	women living in Sirajganj District, prone to flooding	- Out of 64 women who reported problems with harassment, 58% were harassed by their husbands. - 39% were physically abused by their husband		(Based on Mixed Methods Appraisal Tool (MMAT)—out of 5) 40%
Boutilier et al. (2017)	Flood	Calgary, Alberta, Canada 2013 (June 19–July 12)	no. of calls during flood event not recorded	Calls received in Calgary, Alberta related to DV on DV specialized hotline	- 14% increase (6.6 calls per day) in DV related calls in June 2013 from—surrounding neighborhoods experiencing flood conditions		4 out of 8 (50%)
Bradley and Martin (2021)	Earthquake and Floods	Kathmandu and Morgan districts, Nepal Earthquake: April and May 2015 Floods 2017	53 interviews 880 survey participants	Those residing in affected areas	- 2/3 participants reported IPV as most common form of IPV - 36% of 720 female respondents reported IPV and 40% of male respondents reported committing IPV - 14% women reported sexual violence in last 12 months	Qualitative research suggested partner alcohol consumption contributed to violence	30% based on MMAT criteria.
Breetzke et al. (2018)	Earthquakes	Canterbury, NZ Earthquakes September 2010–February 2011. Two most significant events—Darfield (4 September 2010) and Christchurch (22 February 2011) Earthquakes	37,087 police reports; 2 year pre-earthquake (2008–2010) *n* = 20,423, 2 year post earthquake (2011–2013) *n* = 16,664	Residents of New Zealand	- DV only crime to have increased post quake. Approximately 65% of neighborhoods experienced an increase in DV rates post- quake. - DV comprised less than 2% of the total percentage of crimes investigated in this study pre-earthquake; rising to just under 4% post earthquake.	- Most prevalent reports Friday–Sunday - Reported alongside assault	DV only measured based on police reports 4 out of 8 (50%)
Buttell and Carney (2009)	Hurricane (Katrina)	New Orleans, USA, 2005	411,603 calls annually (3 years average)	Residents of New Orleans	- Approximately 13,131 DV related calls p/y prior to Hurricane, reduced to 4,852 calls 1 year post (due to 54% reduction in population) - Rise from 27% to 34.4% post hurricane of average number of DV arrests as a % of the DV calls pre hurricane.		6 out of 8 (75%)
Campbell et al. (2016)	Earthquake	Port au Prince, Haiti, 2010	208	Internally displaced Haitian women, 18–44 years, attending local hospital or clinic	- ¾ reported being abused physically, psychologically, or sexually in the 2-years pre-earthquake (71.2%); and in the 2-years post earthquake (75.0%), 6.9% and 6.6% of these reports were abuse not in context of IPV, respectively - The prevalence of abuse in all categories was not significantly different pre and post earthquake	- Community has limited involvement in safety of women - Abusive partner unemployed - Poor health status - Post Trauma Stress Disorder	100%
Chan and Zhang (2011)	Earthquake	Sichuan Provence, China, May 12, 2008	186	Women over 18 years living in temporary shelter post quake, married/de facto or have children.	- Psychological aggression perpetrated by (ex) spouse increased from 10.5% pre earthquake to 19.3% post earthquake, where spouse or ex-spouse represented 67.9% and 37.1% of the sample, respectively - Pre-earthquake prevalence of physical violence was 5.0%, increasing to 6.6% post quake, where spouse or ex-spouse represented 77.8% and 33.3% of the sample, respectively	Lower physical and mental health	90%
Cools et al. (2020)	Drought and flood (rainfall shocks)	Sub-Saharan Africa (17 countries). 2003–2013	149,032 women in 17 countries (50,512 women in 3rd analysis)	Women 15–49 years	- Statistical analysis suggests no association between IPV and drought periods across 17 countries		100%
Cooper et al. (2021)	Drought	Multiple countries in SSA, LAC, and Asia 2000–2018	363,428 women from 40 countries	Participants in DHS from sub-Saharan Africa, Latin American and Caribbean, and Asia	- No significant relationship between any level of drought and IPV - Strong association with controlling behavior on all three continents - Extreme drought had protective effect on controlling behavior in SSA		100%
Epstein et al. (2020)	Drought	19 countries in sub-Saharan Africa 2011–2018	83,990	Women 15–49 years, married or cohabitating	- Drought associated with IPV toward women (adolescent girls and unemployed women largest association) - Higher risk of women living in severe drought with reports of IPV compared with women not experiencing drought - Women living in mild/moderate drought had higher risk of IPV than those not living in drought. - No association between drought and emotional abuse identified	- Adolescent - Unemployed	100%
Fothergill (1999)	Flood	Grand Forks, North Dakota, USA April, 1997	53 protection orders Two women interviewed	Women living in Grand Forks area, requesting protection order due to DV situations	- Prior to flood (January to March 1997): 20 protection orders issued - Post flood (January to March 1998): 33 protection orders issued - Physical DV began 1 year after flood	- Emotional DV- Previous abuse from childhood - Low-socio, lack of resources - Physical disability	10 out of 10 (100%)
Frasier et al. (2004)	Hurricane (Floyd) and flood	North Carolina, USA. September, 1999	785	Women over 18 years, employed in 12 “blue collar” worksites	- No interaction between IPV and flood was observed, but short time frame measured post-flood - Findings also suggest that women who have previously experienced IPV were 25% more likely to report being affected by the hurricane and floods than women who had not reported IPV in the past	- IPV related to greater stress, PTSD symptoms, and somatic and psychological issues.	100%
Harville et al. (2011)	Hurricane (Katrina)	Tulane Lakeside Hospital, Metairie, LA, and Women’s Hospital, Baton Rouge, LA, USA August 29, 2005	123	6 months postpartum women (gave birth between March 2006 and May 2007)	- Strong relative risks for relationship between damage due to storm and physical or psychological IPV - Experiencing damage due to storm associated with increased likelihood of most conflict tactics. - Experiences associated with hurricane lead to increased likelihood of violent methods of conflict resolution	- Mental health - Daily stress	100%
Houghton (2009)	Floods, snowstorm	Lower Hutt, Masterton, Palmerston North/Fielding, Timaru, Whakatane, NZ February 2004, June 2006, July 2004	54 women month prior; 77 women month of event Five Case studies of Women’s refuge services	Women reporting to a women’s refuge in the month prior and month of disaster event Agencies that support DV during crisis	- Study reported that women’s refuges experienced increase in DV reporting, increased figures in the month of the disaster compared to prior. - Increase in DV related police reports up to 6 months post floods		90%
Houghton et al. (2010)	Snow storm	Ashburton, Waimate and Timaru Districts, New Zealand June, 2006	Exact no. of reports unknown Seven interviews	Women residing in affected area and reporting the Women’s Refuge	- Increase of DV reports to police according to case file summaries by the Women’s refuge, almost double the monthly intake (avg. 14 p/m to 25 p/m) in July 2006 post snow storm - Initial decrease in reports due to phone lines affected by snow storm immediately after event - 57.2% of women were first time reporters - 65.9% had experienced abuse between 2 and 10 years	None of agencies had emergency management plan or policy in place Safe houses were cut-off to services incl. food	100%
Larrance et al. (2007)	Hurricane (Katrina)	Mississippi and Louisiana August 29, 2005	366 IDP’s (171 male, 195 female)	Residing in Federal Emergency Management Agency trailer parks post Hurricane	- Rates of IPV post displacement three times higher than U.S. baseline rates - IPV rape 16 times higher than yearly rate. - Lifetime rate of IPV of sig. 25%	Major depressive disorder Suicide ideation and attempts	8 out of 8 (100%)
Picardo et al. (2010)	Hurricane (Katrina)	Louisiana, USA August 29, 2005	66	English speaking women 18–49 residing in FEMA housing	- 23% of women reported verbal threats from partner, compared to year before hurricane with the same partner - 13% women reported new physical abuse, 33% increased abuse, 20% same amount of abuse, 13% reported decreased abuse with same partner - 20% reported new abuse with a new partner. - 6% forced to have sex since hurricane, 2% declined to answer		5 out of 8 (60%)
Rai et al. (2021).	Drought (2015–2016),	Drought—10 states, cyclones four states across India Drought (2015–2016), cyclones Phailin (2013) and Hudhud (2014)	8,469	Ever-married women who participated in the National Family Health Survey aged 15–49 years	- Positive association between cyclone and three IPV forms. - Women who were residing in districts affected by cyclones had 59% higher odds of emotional violence - Higher odds of experiencing physical and sexual IPV compared to those who had not - No association identified between drought and IPV.	- Reported lower income household - Low education level - History of husband’s alcohol consumption	100%
Rao (2020)	Tsunami	Tamil Nadu, Kerala, Andhra Pradesh, Karnataka December 24, 2004	1999: 3,973 2006: 12,912 2016 12,912	Women aged 15–49 who participated in National Family Health Survey in 1999, 2006 and 2015–2016 (ever-married women in DV module)	- IPV increase in all states except Kerala 2005–2015 - 1998–1999: 90% increased odds of IPV in Tamil Nadu, 46% reported physical violence in the past year - 2005–2006: Highest rates of all IPV in Tamil Nadu (27.9%) and Andhra Pradash (26.1%) 2015–2016: Tamil Nadu (39.33% all IPV) and Andra Pradash (37.14% all IPV) were most affected by tsunami and had highest rates of IPV	- Low education (2005 grp) - Partner low education. (2005 grp) - Member disadvantaged caste group (1999 grp) - Low income house (all grps) - Belonging to minority religion (2005 grp) - Partner consumption of alcohol (all groups) - Living in urban areas (2015–2016 grp)	100%
Sakurai et al. (2017)	Great East Japan Earthquake	Miyagi Prefecture (inland, north coast, south coast), Japan March 11, 2011	7,600	Pregnant women from the Miyagi Unit Center	- Incidence of mental DV in north coast (15.7%), inland (15.2%) and south (18.8%) higher than national average of 13.8% - Study did not identify a sig. incidence of mental DV in the 3–6 month period post disaster. - Physical DV higher in northern coastal area at 5.9% than nationwide or inland areas	- Stress associated with loss of loved one (20.1%) - Experiencing disease or injury of someone close - Changes in family structure - Psychological stress associated with mental DV	7 out 8 If it has not been measured by a validated scale is that ok? (90%)
Schumacher et al. (2010)	Hurricane (Hurricane Katrina)	Mississippi, USA 29-August-05	445	adults 18+ (male and female) either cohabiting (29) or married (416)	- 35% increase in the prevalence of psychological IPV against women pre to post hurricane - 98% increase in prevalence of physical IPV against women from pre to post hurricane	- Young - Married - High school incomplete - Reported pre psychological victimization associated with increased risk for post psychological victimization. - Increased risk of physical IPV among women who experience large number of hurricane related stressors	7 out of 8 If it has not been measured by a validated scale is that ok? (90%)
Tanoue et al. (2021)	Great East Japan Earthquake	Miyagi Prefecture, Japan March 11, 2011	79,222	Pregnant women in the Japan Environment and Children’s Study	- Increase of mental and physical IPV immediately after earthquake in Miyagi Prefecture - Inland areas had highest physical and mental IPV. Highest odds of mental IPV in 2011. - Sustained mental and physical IPV in the inland area, coastal victims received more support post disaster as opposed to inland		8 out of 8 (100%) If it has not been measured by a validated scale is that ok?
Weitzman and Behrman (2016)	Earthquake	Haiti, inc. displacement camps January 12, 2010	2005–2006: 2,535 2012: 6,287	Women in affected areas of Haiti who participated in (DHS)	- Women living in most affected area had higher statistical probability of physical and sexual violence 1–2 years after earthquake (9.3% and 2% higher probability, respectively). - Increased men’s controlling behavior. - IPV higher in women residing in displacement camp	- Reduced employment in women post quake - Loss of access to social services - Those who had lost family - Affected by cholera outbreak	7 out of 8 (90%)
Westhoff et al. (2008)	Hurricane	Refugee camps in Southern Belize October 22–November 2, 1998	202 total; 121 refugees (55.4% men, 44.6% women) 81 displaced people from local health clinic	Residing in a refugee camp and local care health clinic	- 29.6% reported they worried a lot about nonconsensual sex. - 37.2% responded to experiencing sexual violence in the camps (33.3% husband/friend) - 13.3% reported physical violence - 60% of those respondents report physical violence by their husbands		6 out of 8 (75%)

**Table 3.** Risk and Protective Factors in Natural Disasters.

Type of Event	Risk Factors	Protective Factors: Practice and Policy Suggestions
Droughts	- Adolescent women and girls (Epstein, et al., 2020) - Women aged between 15 and 49 (Cools et al., 2020) - From lower socio-economic households; alcohol consumption and/or alcohol abuse (by the perpetrator); lower education levels; (Cooper et al., 2021) Unemployment; increased stress due to insecure resources; loss of access to secure resources; undernourishment and poverty affecting ability for self-control (of the perpetrator); food insecurity; financial strain on the household; mental health conditions, especially depression (Rai et al., 2021)	- Minimize or ban alcohol post disaster and/or during drought - Offer education and job opportunities for men and women post disaster Increase funding and access to emergency income to support families from lower socioeconomic backgrounds post disaster
Earthquakes	- Most prevalent calls made to police Friday–Sunday, indicating possible alcohol use (Breetzke et al., 2018) - Post-disaster stress (Chan & Zhang, 2011) Changes in family functioning; the household economy; women’s access to social network; displacement (Weitzman & Behrman, 2016).	- Integrate violence prevention strategies into post-disaster interventions (Campbell et al., 2016; Chang & Zhang, 2011) - Minimize or ban alcohol post disaster; increase funding and access to emergency income to support families from lower socioeconomic backgrounds post disaster (Weitzman & Behrman, 2016) Provide services and support to help monitor pregnant women; offer sexual health and prenatal services post disaster (Sakurai et al., 2017)
Earthquakes and floods	Alcohol use; stress; poverty; cultural forms of discrimination; lack of privacy in temporary housing conditions and camps due to displacement after homes being destroyed (Bradley & Martin, 2021)	- Include specialist women’s organizations and advisors, funded to support a reduction in risk and vulnerabilities for women post-disaster (Bradley & Martin, 2021) - Construct temporary shelters that ensure women are not living in close proximity to men from other households; toilets need to be gender-segregated and well-lit (Bradley & Martin, 2021) - Minimize or ban alcohol post disaster - Offer education and job opportunities for men and women post disaster Increase funding and access to emergency income to support families from lower socioeconomic backgrounds post disaster
Floods	Alcohol consumption by the partner; lower socioeconomic status of the household; disability; lack of access to services; heavy drinking by perpetrator; partner working in agriculture (Allen et al., 2021)	- Support women to develop their own mitigation and adaptation strategies to reduce flood risks and rebuild homes, protect property, and ensure livelihood security (Allen et al., 2021; Azad et al., 2014). - Provide laws and legal support to prevent violence against women; develop a market system that ensures profits can be generated and essentials can be purchased by women at affordable prices (Boutilier et al., 2017) - Introduce local and home-based industries; flood resistant crops and the distribution of these seeds in flood prone areas can be introduced with special emphasis on women’s home-based crop cultivation (Boutilier et al., 2017) - Plan and implement adequate flood forecasting, warning systems, and preparedness programs (Boutilier et al., 2017) - Provide gender-friendly toilet facilities such as separate toilets at shelter centers (Boutilier et al., 2017) - Provide financial supports to rear cattle and engage women in other income-generating activities (Boutilier et al., 2017) Increase publicly funded childcare and affordable family outings; work with sporting organizations to better educate and support gender equity, healthy relationship skills and bystander skills; increase training in social and emotional learning for parents and families; and conduct further research on the role of alcohol in domestic violence (DV); services such as emergency shelters and crisis counseling (Fothergill, 1999)
Floods, snowstorms	Women reporting to a women’s shelter	Factor in an increase in DV after natural disasters in disaster management (Houghton, 2009).
Hurricanes	- Residing in a refugee camp and married - Displacement and loss of community - Declining mental health - Lack of education - Women of color - Likelihood of intimate partner violence (IPV) increased with age from 18 to 36 and decreased from 37 to 85 - 6 months postpartum women Lower economic status and alcohol use by the perpetrator	- Create access to community services and mental health support post disaster (Anastario et al., 2008; Schumacher et al., 2010) - Incorporate plans to support those affected by IPV post disaster (Anastario et al., 2009) - Increase police presence post disaster (Buttell & Carney, 2009) - Provide education around increased risk of IPV post disaster for services within the community (Harville et al., 2011) - Provide stable housing as a priority for infrastructure focus post disaster (Larrance et al., 2007) - Support community-driven sanctions and interventions around IPV and the establishment of safe havens (Picardo et al., 2010) - Provide access to medical services and education about reproductive health issues are needed following disasters (Westhoff et al., 2008) Create women’s specific health services (for support with DV services, maternal services, family and community connection and social support groups etc.)
Hurricanes and floods	- Women over 18 years and employed in “blue collar” jobs Increased stress, PTSD, negative coping strategies (Frasier et al., 2004)	- Provide access to community services and mental health support post disaster - Minimize or ban alcohol post disaster Provide displacement camps and accommodation with options to be separated by sex/gender (offering safe spaces for women)
Snowstorms	Women residing in affected area and reporting to the local women’s refuge	- provide services during and post disasters, including women’s safe houses and refuges, along with other key emergency support (Houghton et al., 2010) Include planning and policies specific to DV in disaster policy and management plans, along with other critical emergency support such as food and other supplies (Houghton et al., 2010)
Tsunamis	Lower education (of women and/or partner); disadvantaged caste group; lower income households; belonging to a minority religious group; partner alcohol consumption; living in an urban area; belonging to disadvantaged group (Rao, 2020)	- Increase resources and services to target those with socio-economic and demographic vulnerabilities to mitigate further risk of IPV (Rao, 2020) - Implement emergency management plans and policies for disadvantaged areas prone to natural disasters Increase funding and access to emergency income to support families from lower socioeconomic backgrounds post disaster

Out of the 22 studies (*n* = 22 of 27) that were appraised with the quantitative critical appraisal tool, 20 scored 60% or higher (see Table 2). Of those that scored below 60%, this was primarily due to inadequate measurement of IPV in relation to study aims or relevance of the study’s research questions for this review. Therefore, the score may not be representative of the whole study. The three case studies that were appraised with the qualitative tool scored 90% or above. The mixed-methods studies received a score of 40% and 30% due to not providing a method of statistical analysis. The MMAT criteria stipulate that the overall score cannot exceed a score given for each method (Hong et al., 2018).

### Theoretical Frameworks Adopted

Most studies (20 out of 27, or 74%) did not explicitly use a theoretical framework. Of the seven articles that applied a theoretical framework, the key frameworks were the General Affective Aggression Model (Boutilier et al., 2017), Social Disorganization Theory (Shaw & McKay, 1942), Routine Activity Theory (Breetzke et al., 2018; Cohen & Felson, 1979), feminism (Buttell & Carney, 2009; Walker, 1984), a social psychological perspective (Weitzman & Behrman, 2016), vulnerability theory (Rao, 2020), universal risk theories (Fothergill, 1999), and a global humanitarian aid model (Larrance et al., 2007).

### Disaster Type and Countries

As shown in Table 2, a range of disaster types and countries were explored. The most common disaster studies were hurricanes and cyclones. Nine studies examined IPV after a hurricane, six of these examined the impacts of Hurricane Katrina or Rita in multiple locations across the United States (Anastario et al., 2009; Anastario et al., 2008; Buttell & Carney, 2009; Harville et al., 2011; Picardo et al., 2010; Schumacher et al., 2010). One study took place in South Belize post Hurricane Mitch (Westhoff et al., 2008) and another in the United States, Hurricane Floyd (Frasier et al., 2004). Two cyclone events in India were included within the same study, Cyclones Phailin and Hudhud (Rai et al., 2021).

Floods were included in six studies examining IPV. These included flood events in Bangladesh (Azad et al., 2014), sub-Saharan Africa (Cools et al., 2020), New Zealand (Houghton, 2009), Canada (Boutilier et al., 2017), and North Dakota (Fothergill, 1999; Frasier et al., 2004). A further four studies examined the relationship between droughts and IPV in sub-Saharan Africa (Cools et al., 2020; Cooper et al., 2021; Epstein et al., 2020), India (Rai et al., 2021), and Latin America and Asia (Cooper et al., 2021).

Six studies examined IPV after an earthquake, with two studies focusing on the Great East Japan Earthquake in the Miyagi Prefecture (Sakurai et al., 2017; Tanoue et al., 2021), two studies in Haiti (Campbell et al., 2016; Weitzman & Behrman, 2016), multiple earthquakes in the Canterbury region in New Zealand (Breetzke et al., 2018), and one earthquake in the Sichuan Provence, China (Chan & Zhang, 2011).

Two studies included a snowstorm in New Zealand (Houghton, 2009; Houghton et al., 2010). One study included a tsunami across four states in south India (Rao, 2020).

### Types of Measures of IPV

Six studies indirectly measured IPV, using reports from support workers in a women’s refuge (Houghton, 2009; Houghton et al., 2010), protection orders issued by a community violence intervention center (Fothergill, 1999), and police reports of DV (Boutilier et al., 2017; Breetzke et al., 2018; Buttell & Carney, 2009). Five studies measured IPV based on questions developed by the authors and administered as a verbal questionnaire. Two of these measured sexual and physical violence (Anastario et al., 2008; Picardo et al., 2010), one measured sexual violence only (Westhoff et al., 2008), one measured physical and psychological IPV (Schumacher et al., 2010), and the fifth measured IPV as lifetime experience and since displacement (Anastario et al., 2009). One study measured mental, physical, and sexual “harassment” via a verbal interview and questionnaire (Azad et al., 2014).

There was no consistent measure of IPV in the identified studies. Most studies included more than just measures of physical IPV. About one-third of the studies relied on existing self-report measures. Five studies (Campbell et al., 2016; (Chan & Zhang, 2011); Frasier et al., 2004; Harville et al., 2011; Cools et al., 2020) used validated and reliable self-report survey tools to measure physical, sexual, and emotional IPV, such as the Women’s Experience with Battering scale, Severity of Violence Against Women Scale, Danger Assessment, and the Miller Abuse Physical Symptom and Injury Scale. Two studies utilized the Abuse Assessment Screen to measure physical and psychological violence (Chan & Zhang, 2011; Frasier et al., 2004). Two studies utilized the Conflict Tactics Scale (Cools et al., 2020; Harville et al., 2011).

Only nine studies used population-based survey data. Data from the Demographic and Health Surveys (DHS) in sub-Saharan Africa, Haiti, Latin America, and Asia were used in five studies (Allen at al., 2021; Cools et al., 2020; Cooper et al., 2021; Epstein et al., 2020; Weitzman & Behrman, 2016). Two studies relied on data from the National Family Health Survey in India (Rai et al., 2021; Rao, 2020). Two studies used the Japan Environment and Children’s Study (Sakurai et al., 2017; Tanoue et al., 2021).

### Association Between Natural Disasters and IPV

#### Physical IPV

Out of the 17 studies that reported results on the impact of exposure to a natural disaster on physical violence toward a spouse or partner, 14 (82%) identified a significant increase in physical violence post disaster (Allen et al., 2021; Azad et al., 2014; Campbell et al., 2016; Chan & Zhang, 2011; Epstein et al., 2020; Fothergill, 1999; Harville et al., 2011; Picardo et al., 2010; Rao, 2020; Sakurai et al., 2017; Schumacher et al., 2010; Tanoue et al., 2021; Weitzman & Behrman, 2016; Westhoff et al., 2008).

Notably, pregnant women affected in the northern coastal area of the Miyagi prefecture after the Great East Japan Earthquake experienced a 5.9% increase of physical DV, post 3–6 months, higher than the other exposed areas and the national average (Sakurai et al., 2017, *n* = 7,600). In a long-term follow-up of the same disaster in the same locations, a decrease in physical IPV over 2 years was found, based on data from 2011, 2012, and 2013 (Tanoue et al., 2021, *n* = 79,222), suggesting that physical DV spiked immediately post earthquake, but decreased in the years following. However, physical IPV in the inland areas was *sustained* 2 years post disaster (Tanoue et al., 2021). Similarly, women living in displacement camps of the most devastated areas post the Haiti earthquake had a 9.3% higher chance of experiencing physical IPV than those in less affected areas (Weitzman & Behrman, 2016, *n* = 2,535 {2005–2006}, *n* = 6,287 {2012}). An association between physical IPV and exposure to a tsunami was also found, with prevalence of physical IPV doubling in Tamil Nadu, Andhra Pradesh, and Karnataka over the 10 years post the Boxing Day Tsunami (Rao, 2020, *n* = 12,912).

In contrast, exposure to drought does not appear to be significantly associated with physical IPV in sub-Saharan Africa (Cools et al., 2020, *n* = 149,032; Cooper et al., 2021 *n* = 194,820), India (Rai et al., 2021, *n* = 8,469), Asia (*n* = 100,647), or Latin America (*n* = 67,961). However, one study of drought in Africa suggests that the risks of experiencing physical violence may be higher for women living in extreme droughts, compared to mild/moderate droughts in Africa (Epstein et al., 2020).

#### Emotional and Psychological IPV

Out of the ten studies that reported on psychological IPV, seven (70%) identified a significant increase post disaster (Chan & Zhang, 2011; Harville et al., 2011; Picardo et al., 2010; Rai et al., 2021; Sakurai et al., 2017; Schumacher et al., 2010; Tanoue et al., 2021). Three studies (Epstein et al., 2020, *n* = 83,990; Rai et al., 2021, *n* = 84,69l; Frasier et al., 2004, *n* = 785) did not identify an association between natural disasters and psychological IPV.

Two studies on the Great East Japan Earthquake had contrasting results, where Tanoue et al. (2021, *n* = 79,222) identified an increase in psychological IPV immediately after the earthquake, with prevalence decreasing over the following 2 years in all parts of the Miyagi Prefecture, except the northern coastal area. In contrast, Sakurai et al. (2017, *n* = 7,600) identified no increase in psychological DV in the 3–6 months post disaster. However, all areas in the Miyagi prefecture had a higher rate of psychological DV compared to the rest of the nation. Psychological DV was also significantly associated with disease, injury, and changes in the family structure. Emotional IPV was also associated with those residing in the most affected areas post cyclones across 10 states in India, with 59% higher odds of experiencing emotional IPV (Rai et al., 2021, *n* = 8,469). One study did not identify an increase in emotional abuse post the 2010 Haitian earthquake (Campbell et al., 2016, p. 9, *n* = 208); however, the rates of abuse were already “unacceptably high” with three quarters of the study population reporting abuse in the 2 years pre-earthquake.

#### Sexual IPV

Out of the 12 studies that reported sexual violence, eight (66%) identified an association between disaster and sexual IPV (Anastario et al., 2008; Epstein et al., 2020; Picardo et al., 2010; Rai et al., 2021; Rao, 2020; Weitzman & Behrman, 2016; Westhoff et al., 2008). Due to sensitivity, it should be noted that sexual violence is likely to be under reported in these studies. Chang and Zhang (2011)’s study in China, for example, did not measure sexual IPV due to cultural sensitivity.

Rai et al. (2021, *n* = 8,469) identified a high probability of women experiencing sexual violence in areas affected by a cyclone in India. Post tsunami in India, sexual violence increased between 67% and 231% in the decade after the disaster, in all states except Kerala (Rao, 2020, *n* = 12,912). Bradley and Martin (2021) identified an increased prevalence of sexual IPV in their study in comparison to data from the DHS prior to the 2015 earthquake and 2017 floods in Nepal. However, slower onset disasters (such as droughts) may not result in increased sexual IPV. Three studies did not identify a significant association between sexual IPV and drought in 17 countries in sub-Saharan Africa (Cools et al., 2020, *n* = 149,032; Cooper et al., 2021, *n* = 194,820), India (Rai et al., 2021, *n* = 8,469), Latin America (*n* = 67,961), or Asia (*n* = 100,647) (Cooper et al., 2021).

Displacement after a disaster presents a significant risk factor for women’s likely experience of sexual IPV. Four out of the seven studies that reported a significant increase in sexual violence occurred within displacement camps (Anastario et al., 2009; Picardo et al., 2010; Weitzman & Behrman, 2016; Westhoff et al., 2008). Women living in displacement camps of the most affected area of Haiti had a 2% statistically higher probability of sexual violence up to 2 years post disaster (Weitzman & Behrman, 2016, *n* = 2,535 {2005–2006}, 6,287 {2012}). In South Belize refugee camps, 33% of women reported sexual violence by a partner/friend (Westhoff et al., 2008, *n* = 202).

### Significant Risk Factors

Studies that controlled for other factors associated with IPV and disaster identified risk factors that were exasperated by the experience of the disaster. Across these studies, there was a vast array of factors, including poor physical health, low education, low household income, unemployment, being younger and married, a history of partner’s alcohol consumption, a history of past childhood abuse, being in a displacement camp, being a woman of color, and belonging to a minority religious group. Below, we discuss the risk factors that were the most prevalent in the selected studies.

#### Displacement Camps

Six of the seven included studies on women in displacement camps found an increase in IPV post disaster (Anastario et al., 2008, 2009; Campbell et al., 2016; Chang & Zhang, 2011; Larrance et al., 2007; Picardo et al., 2010; Westhoff et al., 2008). Three studies identified increased prevalence of IPV 1–2 years post disaster within the displacement camps (Anastario et al., 2009, *n* = 420, 5.1% increase 2 years post; Anastario et al., 2008, *n* = 194, 17.5% experience Post-Disaster Gender-Based Violence {PDGB}; Larrance et al., 2007, *n* = 366, 25% lifetime rate of IPV).

Picardo et al.’s (2010) study of the impact of Hurricane Katrina in Louisiana gives particular insight into the effect of displacement on IPV during and after a disaster. Of the 66 women who were interviewed and surveyed in FEMA housing (temporary housing post disaster), 20% reported new abuse with a new partner, 13% reported new physical abuse, 33% indicated increased physical abuse, 20% the same amount of abuse, and 13% reported decreased abuse with the same partner in comparison to pre-hurricane (Picardo et al., 2010). This is supported by similar findings on the impact of Hurricane Katrina in the United States in Anastario et al. (2009) and Larrance et al. (2007), where lifetime IPV increased by 21.9% (2006–2007) and 25%, respectively. It should be noted that women who had not been displaced also experienced a 35% increase in psychological IPV and 98% prevalence of increased physical IPV against women post Hurricane Katrina in Mississippi (Schumacher et al., 2010, *n* = 445). The prevalence of recent IPV increased by 5.1% (Anastario et al., 2009) by internally displaced women living in trailer parks, nearly triple the national yearly rate (Larrance et al., 2007). Despite under reporting and sensitivity around sexual IPV, Larrance et al. (2007) also reported a lifetime rate of intimate partner rape 16 times the national average for women in displacement camps.

#### Mental Health

Mental health was the most prevalent risk factor for women experiencing IPV post disaster. All seven of the studies that controlled for a mental health issue identified a significant relationship, including depressive symptoms (Anastario et al., 2008, 2009; Frasier et al., 2009) and PTSD (Campbell et al., 2016; Frasier et al., 2009). Psychological (Frasier et al., 2009; Sukarai et al., 2017) and daily stress (Harville et al., 2011), due to loss or injury of a loved one (Sakurai et al., 2017; Weitzman & Behrman, 2016), and stress related to the disaster (Schumacher et al., 2010) were also indicators of IPV or increased risk to IPV.

Two studies identified an association between previous psychological IPV to physical IPV (Fothergill, 1999; Schumacher et al., 2010). Schumacher et al. (2010) also identified that women who experienced many hurricane-related stressors were more at risk of post-disaster psychological and physical IPV.

#### Community Resources and Social Support

Five studies identified lack of community or social support and accessibility to resources post disaster a risk factor of increased IPV (Campbell et al., 2016; Fothergill, 1999; Houghton et al., 2010; Weitzman & Behrman, 2016; Tanoue et al., 2021). Women who lacked access to social services also experienced a higher probability of IPV as reported in Haiti (Campbell et al., 2016; Weitzman & Behrman, 2016) and in North Dakota, USA (Fothergill, 1999). This lack of social services, including women’s refuges, would increase the difficulty for women to leave or report IPV.

Furthermore, a study of 79,000 pregnant women in the Miyagi Prefecture post the Great East Japan Earthquake, distinguished between the types of IPV, along with the areas affected by the earthquake, over a period of 2 years (Tanoue et al., 2021). Inland areas had the highest odds of physical and psychological IPV after the Earthquake in 2011, and the authors noted that while the coastal areas were the most affected, they also received the most support as opposed to inland areas. The lack of community resources to assist in the recovery process may lead to increased stress and exacerbate the likelihood of IPV occurrence.

#### Partner Alcohol Consumption

Of the five studies that controlled for partner alcohol consumption, four identified a significant relationship between IPV and partner alcohol consumption (Allen et al., 2021; Bradley & Martin, 2021; Rai et al., 2021; Rao, 2020). One study by Weitzman and Behrman (2016, *n* = 2,535 {2005–2006}, 6,287 {2012}) did not identify alcohol as a risk factor. While these studies did not measure whether alcohol consumption had increased in the partner post disaster, Rao (2020, *n* = 12,912), Allen et al. (2021, *n* = 4,903 {2008}, 4,512 {2014}) and Rai et al. (2021, *n* = 8,469) identified a strong relationship between women affected by disaster, partner alcohol consumption, and all forms of IPV. In comparison to women whose husbands did not drink, the odds were twofold for likelihood of IPV in women whose husbands did drink (Rao, 2020).

Furthermore, Bradley and Martin (2021, *n* = 880) acknowledged partner alcohol consumption, combined with disaster, emphasized the gender disparities among men and women and the role it plays in increased likelihood of IPV. Rai et al. (2021) also acknowledge the cultural aspect of a “failing household” on men’s stress levels in India, post cyclone, and in struggling drought conditions. The authors reported that to combat the emotional stress, alcohol abuse was used as a coping strategy, and alcohol is a known link to IPV. Although these studies do not indicate whether there is a direct link between increased alcohol consumption and disaster, this is likely to be a reasonable intuitive deduction given that it may play a mediating role in increasing the likelihood of IPV. Nevertheless, further research in this area is needed.

## Discussion

Notably, this review found evidence that there is an association between IPV and disasters, across disaster types and countries. In general, our synthesis of the identified studies supports the hypothesis that exposure to natural disasters exacerbates the prevalence of IPV. Of the 27 articles that fit the parameters for this systematic review, 24 of them found a relationship between increased IPV and natural disasters. Some studies investigated multiple forms of IPV, which produced varying results. In summary, 14 out of 17 articles found an increase in physical violence, 7 out of 10 articles documented an increase in psychological IPV, and 8 out of 12 articles reported an increase in sexual violence during and post disasters. Other destabilizing life events may also increase as a result of a natural disaster. In the studies that were part of the review, migration to a displacement camp is a good example of a significant risk factor that increased the risk of IPV post disaster. These risk factors mean that causation cannot be claimed between natural disasters and IPV. Natural disasters magnify risk factors, which compound the effects of women experiencing all types of IPV; therefore, it is not clear whether disasters are a contributing risk factor in and of themselves or an event that triggers and heightens previous risk factors. While causation is inconclusive, it was evident that women were placed at greater risk of danger during and post disasters with an increase in risk factors and various forms of IPV. Parkinson (2019, p. 2355) writes, the question of causation “is less important than acting on the knowledge that increased DV and disasters are linked.” Whether causational or correlational, the association between natural disasters and IPV still requires action from policymakers, service providers, and communities.

Second, it is unknown whether natural disasters escalated already existing IPV or if it leads to new instances of IPV. There were only two studies that differentiated between existing and new violence (Houghton et al., 2010; Picardo et al., 2010). However, these two studies had methodological limitations. Picardo et al. (2010) had limited statistical analysis and methodology, while Houghton et al. (2010) relied on police reports with limited demographic data on reporters. While outside the scope of this systematic review as qualitative study, Parkinson’s (2019) inquiry into the Black Saturday bushfires in Australia found that there was an increase in both already existing IPV and new IPV. However, it is rare for a study to investigate whether disasters can result in new cases of IPV. This gap represents a key limitation of the literature and a key question for future research. A better understanding would be transformative to how social services structure prevention and support services during and post disaster.

Third, exposure to earthquakes and hurricanes appears to have the strongest impact on IPV; however, researchers have given significant attention to the impact of earthquakes and hurricanes, which may represent a sampling bias. For example, in this systematic review, seven studies alone (25% of 27) were focused on a single disaster (Hurricane Katrina). Based on the included studies, there does not seem to be a relationship between drought and IPV (Cools et al., 2020; Cooper et al., 2021; Rai et al., 2021). However, there are gaps within the literature examining “slow disasters,” such as droughts and extreme temperatures, and this review did not consider biological and human-caused disasters. The COVID-19 pandemic created acute disaster like conditions with lockdowns and loss of life, yet over time the impacts have continued even if more contained. In this context, the pandemic has continued to impact substantially on mental health and human functioning (Pfefferbaum & North, 2020) as well as impact on family functioning (O’Leary & Tsui, 2021). Some studies report increased interpersonal and gendered violence during the pandemic often linked to sustained periods in lockdowns (Olding et al., 2021). Slow disasters are often not considered disasters due to their less urgent and dramatic nature; consequently, they do not often get examined in disaster research (Nipperess & Boddy, 2018). The relationship between IPV and slow disasters, such as pandemics, represents another critical area for future research.

Given these findings, it is important to highlight possible practical interventions to assist populations grappling with disasters, as these interventions have the potential to reduce the risk of IPV. Significantly, natural disasters are known to cause decline in mental health, along with symptoms of PTSD, anxiety, depression, suicidal thoughts, and increased substance use (Gearhart et al., 2018). There are increased risk factors for women, children, and adolescents, along with other risk factors such as age, disability, ethnicity, and economic status (Gearhart et al., 2018). Therefore, increasing mental health support and access to sexual health and prenatal services will help form protective factors for women affected by disasters (Fothergill, 1999; Sakurai, et al., 2017). Protective factors include internal factors such as individual hope, religious belief, and optimism, along with external factors such as material and social resources (Gearhart et al., 2018). This understanding can help guide post-disaster interventions by practitioners, who can nurture these important factors in their practice (Gearhart, et al., 2018).

For example, following a natural disaster, to reduce social isolation and declining mental health, service providers must support kinship and family networks to stay intact when communicating with those affected (Lee & First, 2022). However, additionally, it is important that options be available and accessible for same-sex couples, as well as safe accommodation outside of family and kinship networks. Outside of family and wider networks of community acting as a strong protective factor for mental health, relief organizations often provide aid for the first family member to submit an application for financial or other support, with this member receiving direct aid for a wider family unit to be shared; however, this can be seen as supporting nuclear family units more so than women who live in family units that may not include a man as head of the family (Lee & First, 2022). To support nontraditional family structures, women escaping IPV, and those from lower socioeconomic backgrounds, relief workers under pressure and needing to prioritize distributing aid, must also consider that distributing to wider family networks rather than solely to a nuclear family unit may allow for a more equitable, accessible distribution of relief for women affected by natural disasters, such as displaced women, women with disabilities, as well as older women.

To do so, education for these services around supporting diverse families, along with education on the increased risk of IPV, will increase understanding of why such strategies are important (Campbell et al., 2016; Chang & Zhang, 2011). Socioeconomic and demographic vulnerabilities factor into the risk of IPV after disaster; therefore, an increase of resources and services to target those from vulnerable backgrounds may help to mitigate further risk of IPV and lead to further protective factors following natural disasters (Rao, 2020).

Disaster policy and management plans should include planning and policies specific to DV, along with other critical emergency support such as food and other supplies (Houghton et al., 2010). For women escaping to DV shelters, capacity is often further reduced due to temporary housing or possible damage to accommodation options, lessening the ability for women to escape DV and find shelter elsewhere (Lee & First, 2022). DV agencies, including women’s safe houses and refuges, should continue to provide services during and post disasters, along with other key emergency support (Houghton et al., 2010). Displacement camps and accommodation must, therefore, include options to be separated by sex/gender—therefore increasing the possibility of safe spaces for women. Access to safe shelters, along with separate gender toilet and shower facilities, must be prioritized for women post-natural disaster to decrease barriers for women who are escaping DV and other unsafe living conditions (Bradley & Martin, 2021).

## Limitations

There are limitations to the study. The studies were limited to English-speaking articles. Additionally, the articles included were peer-reviewed only, and therefore, information may have been referenced within this review; however, it did not form part of the study itself. The exclusion criteria for this study included violence on any other vulnerable groups, including young people, older people, or other IPV with other household members, and therefore, this research does not consider other vulnerable groups that are affected by IPV. Importantly, research so far completed has not taken into consideration wider vulnerabilities such as the aging population, people with disabilities, and other minority groups. Therefore, there is further scope for exploring the implications of this research for other vulnerable groups.

Additionally, the study also does not directly address diversity of women from within the inclusion criteria. For example, vulnerable groups of women, such as older women, women of color, women with disabilities and people of a diversity of genders, bodies, and sexualities, have not directly addressed. Consequently, there are gaps in our knowledge of how the impacts of IPV may affect various groups, which has implications for practice.

Furthermore, human-caused disasters were excluded, such as oil spills and nuclear failures, and biological disasters including pandemics, and therefore limited the scope of the review to strictly natural disasters.

## Conclusion

This systematic review found evidence of a clear association between natural disasters and IPV. The studies took place across varying cultures and countries, therefore allowing for practice implementation across varying settings, cultures, environments, and natural disasters. The review also identified gaps in the evidence base. For example, what is the mechanism by which natural disasters impact IPV? It is unclear whether disasters have a direct (causal) impact or they are mediated through increases in other risk factors. Does the exposure to natural disasters result in the occurrence of new cases of IPV? There has been little research disaggregating new cases from existing cases of IPV. What is the impact of disasters that have a slow onset? There was an absence of “slow disasters” in the literature with a high representation of earthquakes and hurricanes. Nonetheless, this systematic review provides support for the hypothesis that exposure to natural disasters increases the occurrence of IPV, at least in the short term. Given the increased frequency and severity of disasters in the current environmental, geopolitical, and military context, we need a better understanding of this relationship, as well as the ways in which risk and protective factors can shape the experience of IPV. Using this knowledge, policymakers, service providers, and communities could better respond during and after disasters to minimize existing risk factors and accentuate protective factors, strengthening community capacity, which, in turn, may reduce IPV.
